# Death of Dr. A. S. Heaton

**Published:** 1882-08-20

**Authors:** 


### *DEATH OF DR. A. S. HEATON, OF DETROIT.*

A private letter conveys to us the sad intelligence of the death of Dr. A. S. Heaton, Professor of Clinical Medicine in the Detroit Medical College. We made the acquaintance of Dr. Heaton and his excellent lady at the time of our visit to the American Medical Association at Richmond in 1881, and was most favorably impressed with him as a gentleman and as a medical man of intelligence and fine attainments in the profession. His death took place on the 9th ult. from disease of the heart. A meeting of the physicians of Detroit was called on the following day in honor of the departed brother, and the following resolutions passed:

WHEREAS, It has pleased Divine Providence to remove, in accordance with the order of nature, our brother physician and townsman, ABRAM S. HEATON,

*Resolved,* That the profession of this city and State has lost one of its most distinguished and valued members, and this community a capable and highly esteemed practitioner.

*Resolved,* That we sympathize with the bereaved family, and with those who have been accustomed for so many years to rely upou his advice and assistance in times of sickness.

Dr. Heaton was in his 54th year, and was an active business man and a progressive practitioner. We tender our sympathy to the wife and family of our deceased friend and brother. Peace to his memory! P.

